# Differences in Net Information Flow and Dynamic Connectivity Metrics Between Physically Active and Inactive Subjects Measured by Functional Near-Infrared Spectroscopy (fNIRS) During a Fatiguing Handgrip Task

**DOI:** 10.3389/fnins.2020.00167

**Published:** 2020-03-10

**Authors:** Elizabeth L. Urquhart, Xinlong Wang, Hanli Liu, Paul J. Fadel, George Alexandrakis

**Affiliations:** ^1^Bioengineering Department, University of Texas at Arlington, Arlington, TX, United States; ^2^Department of Kinesiology, University of Texas at Arlington, Arlington, TX, United States

**Keywords:** fatigue, sensory-motor cortex, directional phase transfer entropy, directional connectivity, functional connectivity variability

## Abstract

Twenty-three young adults (4 Females, 25.13 ± 3.72 years) performed an intermittent maximal handgrip force task using their dominant hand for 20 min (3.5 s squeeze/6.5 s release, 120 blocks) with concurrent cortical activity imaging by functional Near-Infrared Spectroscopy (fNRIS; OMM-3000, Shimadzu Corp., 111 channels). Subjects were grouped as physically active (*n* = 10) or inactive (*n* = 12) based on a questionnaire (active-exercise at least four times a week, inactive- exercise less than two times a week). We explored how motor task fatigue affected the vasomotion-induced oscillations in ΔHbO as measured by fNIRS at each hemodynamic frequency band: endothelial component (0.003–0.02 Hz) associated to microvascular activity, neurogenic component (0.02–0.04 Hz) related to intrinsic neuronal activity, and myogenic component (0.04–0.15 Hz) linked to activity of smooth muscles of arterioles. To help understand how these three neurovascular regulatory mechanisms relate to handgrip task performance we quantified several dynamic fNIRS metrics, including directional phase transfer entropy (dPTE), a computationally efficient and data-driven method used as a marker of information flow between cortical regions, and directional connectivity (DC), a means to compute directionality of information flow between two cortical regions. The relationship between static functional connectivity (SFC) and functional connectivity variability (FCV) was also explored to understand their mutual dependence for each frequency band in the context of handgrip performance as fatigued increased. Our findings ultimately showed differences between subject groups across all fNIRS metrics and hemodynamic frequency bands. These findings imply that physical activity modulates neurovascular control mechanisms at the endogenic, neurogenic, and myogenic frequency bands resulting in delayed fatigue onset and enhanced performance. The dynamic cortical network metrics quantified in this work for young, healthy subjects provides baseline measurements to guide future work on older individuals and persons with impaired cardiovascular health.

## Introduction

Cerebral autoregulation helps maintain a relatively constant oxygen supply to the brain during changes in arterial blood pressure, by maintaining cerebral blood flow (CBF) relatively constant. This occurs via vasoconstriction in response to increased blood pressure and vasodilation in response to decreased blood pressure. Regional CBF (rCBF) is flow-mediated vasodilation from distal to proximal vessels that occurs in activated brain regions which protects downstream microvascular pressure (Querido and Sheel, 2007; Peterson et al., 2011; Perrey, 2013; Tzeng and Ainslie, 2014; Peri-Okonny et al., 2015). There are overlapping regulatory mechanisms of rCBF that have been classified into contiguous ranges of hemodynamic frequencies as endogenic (0.003–0.02 Hz), neurogenic (0.02–0.04 Hz), and myogenic (0.04–0.15 Hz) (Cipolla, 2009; Li et al., 2012). These frequency oscillations reflect the influence of endothelial-related metabolic activity, intrinsic neuronal activity, and myogenic activity of the vascular smooth muscle, respectively (Cipolla, 2009; Li et al., 2012; Huo et al., 2018). Exercise may also modulate the regulatory mechanisms in each frequency band. Exercise is known to improve cardiovascular health (Myers, 2003) and brain health (Peri-Okonny et al., 2015; Mueller et al., 2017). Regular exercise produces beneficial alterations in the brain that maintain or improve cognition (Bosch et al., 2017; Mueller et al., 2017), and promote motor function (Perrey, 2013), known as exercise-dependent neuroplasticity.

The hemodynamic oscillations at these frequency bands can be measured using functional brain mapping using functional magnetic resonance image (fMRI) (Zhang et al., 2015), or functional near-infrared spectroscopy (fNIRS) (Li et al., 2012; Bosch et al., 2017; Andersen et al., 2018; Cao et al., 2018b). FNIRS measures non-invasively the change of oxyhemoglobin (ΔHbO) and deoxyhemoglobin (ΔHb) concentrations resulting from neurovascular coupling secondary to neuronal activation by utilizing light at near-infrared wavelengths (650–1000 nm). It is advantageous because of its relatively lower cost, portability, robustness to motion artifacts, and its higher temporal resolution compared to fMRI (Huppert et al., 2009; Naseer and Hong, 2015).

In recently completed work, we demonstrated interesting temporal evolution patterns for hemodynamic activation and static functional connectivity (SFC) changes that depended on the physical activity levels of subjects while they were trying to maintain maximal handgrip task performance (Urquhart et al., 2019). That work identified that physically active subjects experienced delayed fatigue onset as evident by their greater ability to maintain maximum voluntary contraction (MVC) force accompanied by longer-lasting and more spatially extended activation and SFC patterns in the primary motor (M1), premotor and supplementary motor areas (PMC/SMA) and the prefrontal cortex (PFC) (Urquhart et al., 2019). In contrast to the static networks explored in our previous work, the purpose of this work was to examine changes in dynamic cortical network patterns and how they relate to handgrip task performance for each hemodynamic frequency band, as a function of a subjects’ physical activity level.

In this work we first applied directional phase transfer entropy (dPTE) analysis, a computationally efficient and data-driven method previously used in electroencephalography (EEG) research (Hillebrand et al., 2016), to estimate changes in the direction of information flow during the fatiguing handgrip task. It has been applied recently to fNIRS in one study (Cao et al., 2018b), but dPTE analysis has not been employed, to our knowledge, as yet to explore the effect of fatigue on the brain’s networks. The net direction of information flow, or directional connectivity (DC), was also quantified between regions to better understand motor control regulation under fatiguing conditions (Hillebrand et al., 2016). A simultaneous multimodal neuroimaging study using fNIRS, fMRI, and EEG has previously examined directionality via Wiener-Granger causality on finger movement tasks, but their results were confined to the contralateral (i.e., left) hemisphere only and not broader cortical networks (Anwar et al., 2016). We also explored the relationship between SFC and functional connectivity variability (FCV), which represents spontaneous dynamic fluctuations of connectivity over time (Fong et al., 2019). This was motivated by the fact that SFC is known to increase with task performance (Jiang et al., 2012; Perrey, 2013; Urquhart et al., 2019), while FCV reflects resource availability during demanding tasks (Li et al., 2015; Fong et al., 2019). The aim of this work was to identify differences between subject groups across all of the aforementioned fNIRS metrics and hemodynamic frequency bands. In addition, we aimed to demonstrate the feasibility of using time-dependent fNIRS metrics to help understand how dynamic connectivity regulation of cortical networks relates to performance during a fatiguing motor task.

## Materials and Methods

### Participants

Twenty-three young adults (4 females) between the ages 18 and 30 (mean age 25.13 ± 3.72) participated in this study. All subjects gave prior written informed consent for their participation in this study, which was approved by the Institutional Review Board of the University of Texas at Arlington (IRB# 2018-0686) and performed in accordance with the Declaration of Helsinki. All but two subjects were right-handed, as determined by the Edinburgh handedness scale (Oldfield, 1971). All subjects were free of any neurological or psychiatric disorders (self-reported), and were non-smokers. Subjects self-reported as being physically inactive (*n* = 12, exercising less than twice a week for 30 min of moderately vigorous exercise), or active (*n* = 11, exercising at least four times a week for 30 min of moderately vigorous exercise).

### Experimental Procedures

**FIGURE 1:**
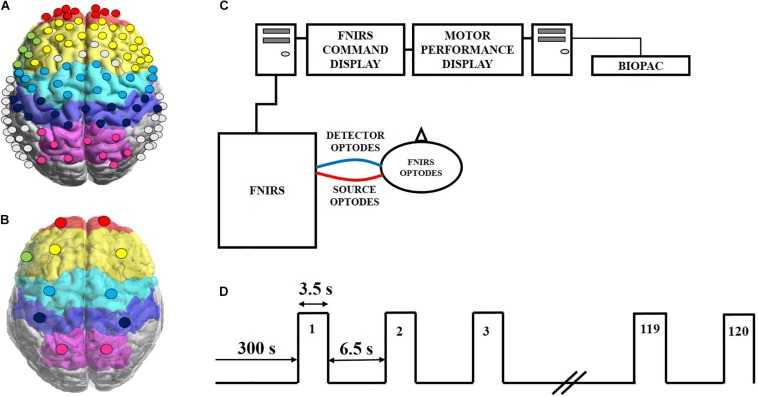
Experimental set up and protocol timeline for the handgrip task. **(A)** FNIRS 111-channel layout with eleven regions of interest (ROIs) covered by the probe geometry: left and right frontopolar (lFP; rFP) (red), left and right pre-frontal cortex (lDLPFC; rDLPFC) (yellow), Broca’s area (green), left and right pre-motor cortex (lPMC; rPMC) (light blue), left and right primary motor and sensory cortical (lM1/S1; rM1/S1) areas (purple), and left and right sensory association cortex (lSAC; rSAC) (pink). **(B)** Each circle shows the spatial average of the probe coordinates in each ROI, per brain hemisphere. These averaged probe locations served as reference points for plotting dPTE and DC between ROIs in this work. **(C)** Schematic of the experimental set-up of the fNIRS (LABNIRS) system and the BIOPAC handgrip force sensor system with one representative source-detector channel shown for simplicity. **(D)** The handgrip task protocol, starting with a 5-minute baseline. Subjects performed intermittent handgrip contractions for 3.5 s followed by 6.5 s of rest for 120 blocks at 100% MVC.

A continuous wave fNIRS imaging system (OMM-3000, Shimadzu Corp., Kyoto, Japan) was used in this experiment, which utilized near infrared light diode sources (780, 805, and 830 nm) and photomultiplier detectors at a sampling frequency of 10.101 Hz. The setup geometry consisted of 32 sources and 34 detectors with a separation of 3 cm, resulting in 111 source-detector channels (Figure 1A). This probe geometry covered cortical areas of the following 11 regions of interest (ROIs) (Figure 1B): left and right frontopolar prefrontal cortex (lFP; rFP), left and right dorsolateral prefrontal cortex (lDLPFC; rDLPFC), Broca’s area, left and right premotor cortex (lPMC; rPMC), left and right primary motor and sensory cortical (lM1/S1; rM1/S1) areas, and left and right sensory association cortex (lSAC; rSAC). Anatomical cranial reference points (nasion, inion, left and right preauricular points and vertex) and optode locations were recorded for each subject using a 3D digitizer (FASTRAK, Polhemus VT, United States). Montreal Neurological Institute (MNI) coordinates for each source and detector locations were calculated using the statistical parametric mapping NIRS_SPM software, which provided the Brodmann area (BA) corresponding to each fNIRS channel as shown in Supplementary Table S1 (Singh et al., 2005).

Subjects sat upright with their dominant arm at their side, elbow flexed at 90° and resting on a table. Subjects faced two screens that displayed protocol commands and visual feedback of handgrip performance (Figure 1C). Prior to starting the handgrip task, subjects performed three to five isometric MVCs with their dominant hand. All MVCs were recorded at a 1 kHz sampling rate using a handgrip dynamometer (BIOPAC, CA, United States). The pre-task MVCs were averaged for each subject and set as their maximum target of 100% MVC to reach during the subsequent handgrip task. The fNIRS data acquisition began with a 5 min resting period where subjects were asked to refrain from any movement or specific thoughts, followed immediately by a fatiguing handgrip task. The task required subjects to perform intermittent handgrip contractions for 3.5 s alternating with 6.5 s of rest for 120 blocks (Figure 1D) while attaining 100% MVC as closely as possible, as a means to induce fatigue in the forearm (Liu et al., 2007; Shibuya and Kuboyama, 2010; Jiang et al., 2012; Rhee and Mehta, 2018). The recorded force time-series data were low-pass filtered at 15 Hz and the maximum force for each block was calculated (Mehta and Shortz, 2014; Rhee and Mehta, 2018).

### Data Preprocessing

This study used the open-source Homer2.0 to process (Supplementary Table S2) the collected fNIRS data (Huppert et al., 2009). Detrending was implemented using the least-squares fit of a line that was subtracted from the data (Xu et al., 2015). The data were then filtered for each respective frequency band. Following previously published work, frequency bands were defined as endogenic (0.003–0.02 Hz), neurogenic (0.02–0.04 Hz), and myogenic (0.04–0.15 Hz) (Aalkjaer et al., 2011; Cao et al., 2018b). Data in each of these three hemodynamic frequency bands were low-pass filtered by a third order and high-pass filtered by a fifth order Butterworth filter (Huppert et al., 2009). Channels were removed if signal standard deviations were greater than two times their mean signal amplitude (Cao et al., 2018b). Principal component analysis (PCA) was utilized to remove motion artifacts and global hemodynamic fluctuations that may overlap with the task-related hemodynamic response frequencies. The first two principal components were removed from all fNIRS channel data in order to remove these global artificats (Huppert et al., 2009; Naseer and Hong, 2015). To avoid signal contamination especially by branches from the middle cerebral artery or the superficial temporal artery and temporal muscle, channels located near these structures were also removed from analysis (Smielewski et al., 1997; Oldag et al., 2012). The resulting optical density data were then converted into changes in hemoglobin concentration relative to baseline (ΔHbO and ΔHb) using the Modified Beer-Lambert Law with an estimated differential pathlength factor of 6.0 for each wavelength, an estimate used in Homer 2.0 (Kohl et al., 1998). Only ΔHbO values were presented in the Result section below because ΔHb values were found to have similar and opposite qualitative trends, but with smaller amplitudes and lower signal-to-noise ratio as previously reported in other neuroimaging studies observing motor activation tasks (Anwar et al., 2013; Visani et al., 2015). Nevertheless, corresponding ΔHb results were included in the Supplementary Material section for completeness. Left-handed subjects’ data was flipped to its mirror image on the brain for group averaging purposes and the subsequent interpretation for all data was right (r) for contralateral and left (l) for ipsilateral brain hemispheres relative to the arm performing the handgrip task.

### Phase Transfer Entropy (PTE) and Directed PTE (dPTE) Data Analysis

The information flow between ROIs was estimated using phase transfer entropy (PTE) based on the same principle as Granger Causality (Siebenhuehner et al., 2013; Hillebrand et al., 2016; Cao et al., 2018b). It is calculated as the difference between the uncertainty of the target signal *Y* conditioned by its past and the uncertainty of the target signal conditioned on both its past and the source signal *X* (Hillebrand et al., 2016; Cao et al., 2018b):

(1) $${\mathrm{PTE}}_{\mathrm{XY}}=H\,\left(Y_{t+\delta }|Y_{t}\right)-H\,\left(Y_{t+\delta }|Y_{t},X\right)$$

where PTE_XY_ is the PTE from source *X* to target signal *Y*. Shannon Entropy (H) is defines as:

(2) $$H\,\left(Y_{t+\delta }\right)=-\sum_{i=0}^{n}p\,\left(Y_{t+\delta_{i}}\right)\,\mathrm{logp}\,\left(Y_{t+\delta_{i}}\right)$$

where the summation is performed for discrete time steps *t* + δ*_i_* (*i* = 0, n), where n signifies the total number of time bins, defined by the product of the time interval duration (rest, or task period) in seconds and the data sampling frequency of 10.101 Hz. The delay between signal *Y*_*t*_ and *Y*_*t*+δ_*i*__ is expressed as δ*_i_*. For a more complete description of how Shannon entropy is computed the reader is referred to relevant prior literature (Shannon, 1948; Schreiber, 2000; Hillebrand et al., 2016).

Due to PTE_XY_ lacking a meaningful upper bound and to reduce bias, a normalizing process is used (Hillebrand et al., 2016):

(3) $${\mathrm{dPTE}}_{\mathrm{XY}}=\frac{{\mathrm{PTE}}_{\mathrm{XY}}}{{\mathrm{PTE}}_{\mathrm{XY}}+{\mathrm{PTE}}_{\mathrm{YX}}}.$$

With a range between 0 and 1, if 0.5 < dPTE_XY_ < 1 the information flow is preferentially from X to Y. But, if 0 < dPTE_XY_ < 0.5, then the information flow is preferentially from *Y* to *X*. In the event that dPTE_XY_ = 0.5, there is no preferential direction of information flow (Hillebrand et al., 2016; Cao et al., 2018b).

### Data Processing Steps for dPTE

Directed PTE analysis was applied to calculate information flow for the endogenic, neurogenic, and myogenic frequency bands at three periods: 5 min of resting state, 0–10 min, and 11–20 min of the handgrip task. Directed PTE for resting state requires a minimum of 5 min to attain stable computed values (Hillebrand et al., 2016) and in this work 10 min periods were used to account for the higher amount of hemodynamic variation during the handgrip paradigm (Urquhart et al., 2019). Firstly, PTE analysis was performed to quantify causality between every two channels among all 111 channels. Then PTE values were normalized into dPTE values, generating a 111 × 111 matrix. The value at Xth row and Yth column determined the scale of information flow from *Y* to *X*. If dPTE was between 0 and <0.5 the net information flow was from *Y* to *X*, whereas if dPTE > 0.5 the net flow was from *X* to *Y*. If dPTE was equal to 0.5, within a rounding error of two decimals, no net information flow was assumed. Then the dPTE was averaged lengthwise by row, yielding a 1 × 111 matrix, which was the mean dPTE between each one channel and all other channels (Cao et al., 2018b). The two channels with the highest ROI percentage overlap relative to neighboring ROIs, as determined by NIRS_SPM, were averaged together and assigned to each ROI, yielding a 1 × 11 matrix. The high signal variability induced during the task, as demonstrated in our previous work (Urquhart et al., 2019), did not allow for identification of statistically significant changes in individual channels. Therefore, the dPTE channels were averaged from up to 11 channels to 2 per ROI in order to allow for comparisons between dPTE at rest and during the task that could lead to statistically significant differences. A one-sample *t*-test was then performed across subjects for significant differences in dPTE across all possible pairs of ROIs (*p* < 0.05 and false discovery rate (FDR) corrected) (Singh and Dan, 2006). This test was used to determine significant net information flow into or out of each ROI by testing against the null hypothesis of no net flow, where positive *t*-values indicated outgoing net information flow (“source”) and negative t-values indicated incoming net information flow (“sink”). Significant net information flow values for each ROI were visualized using BrainNet Viewer, an open-source software package (Xia et al., 2013), for each frequency band at each period per subject group. Dedicated software (G^∗^Power v3.0.10, Franz Fual, Kiel University, Kiel, Germany) was used to perform *post hoc* statistical power (1-β) analysis (Faul et al., 2007).

### Directional Connectivity (DC)

The dPTE values were first averaged to a single channel within each of the ROIs, generating an 11 × 11 matrix. A one-sample *t*-test was then performed across subjects for significant differences in dPTE across all possible pairs of ROIs (*p* < 0.05 and FDR corrected) (Singh and Dan, 2006). This test was used to determine directionality of significant information flow between each pair of ROIs by testing against the null hypothesis of no net flow (dPTE_XY_ = 0.5), where positive *t*-values indicated net information flow from ROI_1_ to ROI_2_ and conversely for negative *t*-values. Topographic images for DC were generated using BrainNet Viewer software (Xia et al., 2013) for each frequency band at each period per subject group.

### Static FC (SFC) and FC Variability (FCV) Analysis

For each subject’s data set, a static functional connectivity (SFC) matrix was generated by computing the Pearson’s correlation coefficient (*r*) between the ROI-averaged channels per brain hemisphere (Li et al., 2015; Fong et al., 2019). Subsequently, dynamic functional connectivity (DFC) between pairs of ROIs was calculated using a sliding-window correlation (SWC) (Allen et al., 2014; Li et al., 2015; Fong et al., 2019). In this study, a 60 s time window was selected and shifted in 1 s increments along the entire time course as described in prior fNIRS studies (Hutchison et al., 2013; Li et al., 2015; Cao et al., 2018a). The FC within each time window was also calculated for each pair of ROIs via the Pearson’s correlation coefficient. Then the functional connectivity variability (FCV) was calculated as the standard deviation of the correlation coefficient along time (Fong et al., 2019). For group analysis, the FCV of each correlation coefficient for each ROI was averaged across subjects in each group.

## Results

### Relative Changes in MVC Over Time

**FIGURE 2:**
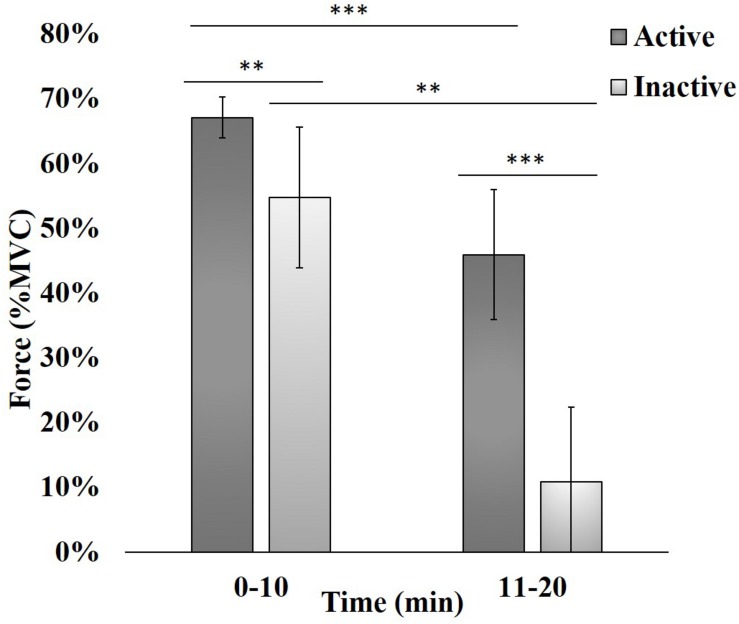
Force produced during intermittent handgrip contractions while physically inactive and active subjects attempted to attain 100% MVC. Each bar represents an average of 60 consecutive trials, expressed as the Mean (bar height) ± Standard Error to the Mean (SEM; error bar). ^∗∗^*p* < 0.01, ^∗∗∗^*p* < 0.001.

The loss of handgrip force, expressed as the relative reduction in %MVC compared to the pre-task maximum value, was quantified as a proxy measure of fatigue (Figure 2). Force data was first averaged over 12 blocks for the 0–2 min period. An independent *t*-test, satisfying normality and equal variance assumptions, determined that there was no significant (*p* > 0.05) difference between groups (not shown), indicating subjects did not fatigue within this initial period. Force data were then averaged over 60 blocks resulting in two periods (0–10 min and 11–20 min) across the 120 contractions. As these data periods did not meet assumptions for independent *t*-tests, data was analyzed using the non-parametric Mann-Whitney *U* test (Marques, 2007). The absolute force produced by active subjects was significantly higher than inactive subjects at 0–10 min (*p* < 0.01) and 11–20 min (*p* < 0.001). Lastly, %MVC force decreased significantly between periods in both active (*p* < 0.001) and inactive (*p* < 0.01) subjects.

### Frequency Band Analysis With Information Flow and Directional Connectivity

The net information flow among the eleven ROIs (lPF, rFP, lDLPFC, rDLPFC, Broca’s, lPMC, rPMC, lM1/S1, rM1/S1, lSAC, and rSAC) during the three task periods (rest, 0–10 min, and 11–20 min) was computed by dPTE analysis for the endogenic, neurogenic, and myogenic hemodynamic frequency bands and plotted in color-coded maps. In these maps, blue ROIs indicate net incoming information flow (the channels at those cortical locations are a “sink”) and red ROIs indicate net outgoing information flow (these channels are “sources”). Green ROIs indicate no statistically significant net flow.

The directionality of information flow (DC) was averaged within each of the eleven ROIs, so as to enable averaged, region-specific dPTE values that were amenable to statistical comparisons. The latter yielded differing spatial DC patterns between inactive and active subjects that are presented here for each hemodynamic frequency band. The unidirectional arrows originate from a source ROI and end in a sink ROI. Black arrows correspond to a significance of *p* < 0.05 and red arrows denote a significance of *p* < 0.01.

All one-sample *t*-tests satisfied normality assumptions for dPTE and DC analyses. While physiological interferences were minimized using band-pass filters and PCA filter for each frequency band, global mean removal was also applied, and results were effectively indistinguishable between the methods. A *post hoc* power analysis with α = 0.05 yielded a statistical power of 80% for both groups.

#### Endogenic Frequency Band

**FIGURE 3:**
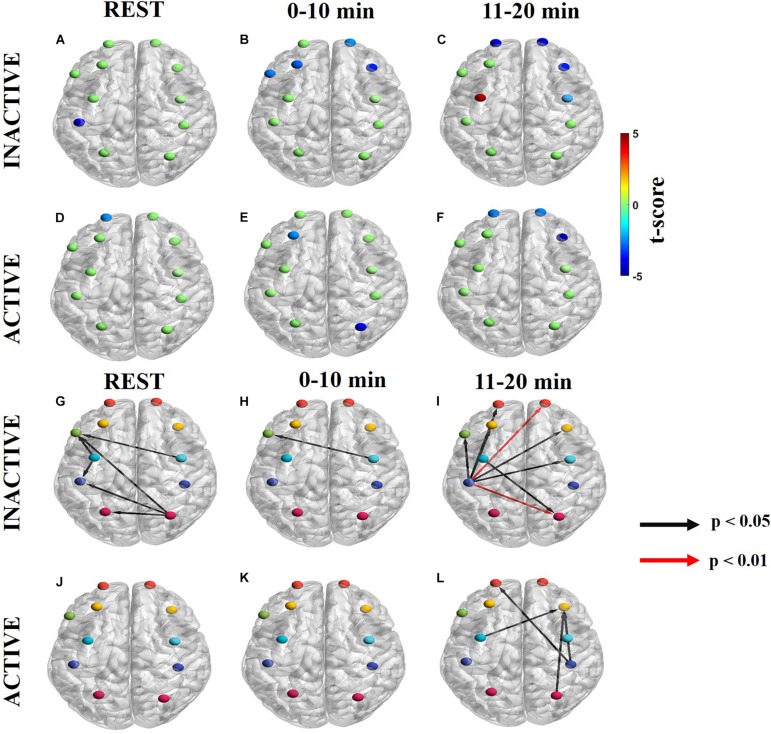
Significant dPTE and DC in the endogenic frequency band for inactive and active subjects during the handgrip task for ΔHbO. Directed PTE *t*-values for each ROI as a color-coded map for inactive subjects **(A–C)** and active subjects **(D–F)**. Hot (yellow-reds) and cold (light blue-dark blue) colors indicate information outflow and inflow, respectively. Arrows indicate statistically significant information flow between functional regions for inactive **(G–I)** and active subjects **(J–L)**. Black arrows (*p* < 0.05); Red arrows (*p* < 0.01). Eleven regions of interest (ROIs) were mapped: left and right frontopolar (lFP; rFP) (red), left and right pre-frontal cortex (lDLPFC; rDLPFC) (yellow), Broca’s area (green), left and right pre-motor cortex (lPMC; rPMC) (light blue), left and right primary motor and sensory cortical (lM1/S1; rM1/S1) areas (purple), and left and right sensory association cortex (lSAC; rSAC) (pink).

Maps of net information flow in the endogenic frequency band are shown in Figures 3A–C (Supplementary Figures S1A–C for ΔHb) for inactive subjects and Figures 3D–F (Supplementary Figures S1D–F for ΔHb) for active subjects. Outgoing information (sources) differed between the groups at all time periods. Inactive subjects had a statistically significant sink at lM1/S1, whereas active subjects had a dPTE sink at lFP at rest in the endogenic frequency band (Figures 3A,D). For inactive subjects, rFP, lDLPFC, rDLPFC, and Broca’s area were statistically significant sinks initially (Figure 3B) that later became more strongly unilateral at rFP, rDLPFC, and rPMC as the task progressed (Figure 3C). Additionally, inactive subjects had a statistically significant dPTE source at lPMC (Figure 3C). In contrast, active subjects had a significant sink at lDLPFC and rSAC initially (Figure 3E) that changed to lFP, rFP, and rDLPFC as the task progressed (Figure 3F).

In the endogenic frequency band, inactive subjects had notably more ROI pairs with significant DC than active subjects at rest (inactive: 6, active: 0), in the 0–10 min (inactive: 1, active: 0), and the 11–20 min intervals of the task (inactive: 8, active: 4) (Figures 3G–L and Supplementary Figures S1G–L for ΔHb). During the task, inactive subjects initially had statistically significant DC form rPMC to Broca’s area (Figure 3H). However, as fatigue worsened in the second half of the task lM1/S1 became the primary functional area source connecting to bilateral FP, bilateral PFC, Broca’s area, rPMC, and rSAC (Figure 3I). Additionally, inactive subjects had two DC pairs in the 11–20 min period (lM1/S1 to rFP and lM1/S1 to rSAC) that had higher statistical significance (*p* < 0.01) (Figure 3I).

In contrast, active subjects had an absence of significant net information flow between ROIs during rest and the 0–10 min periods (Figures 3J,K) and only exhibited significant net information flow between a few functional regions (from lPMC, rM1/S1, and rSAC to rDLPFC and rM1/S1 to lFP) in the 11–20 min period (Figure 3L).

#### Neurogenic Frequency Band

**FIGURE 4:**
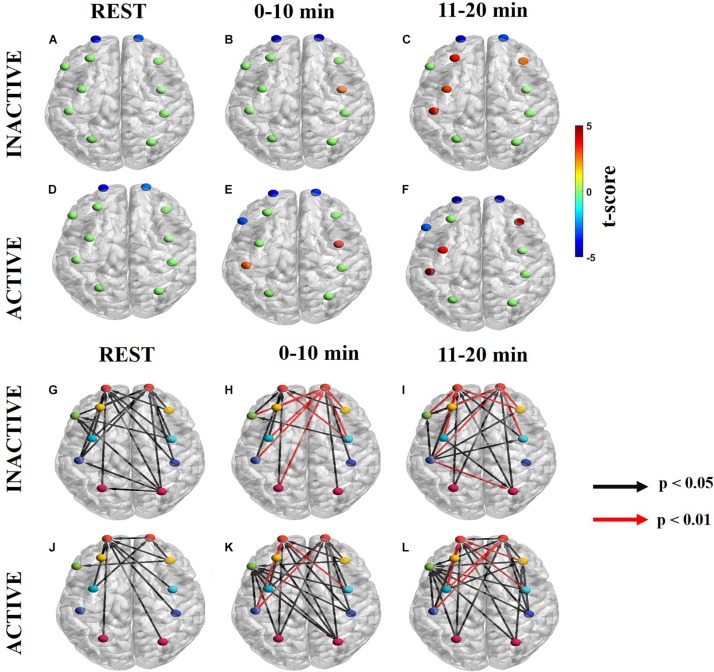
Significant dPTE and DC in the neurogenic frequency band for inactive and active subjects during the handgrip task for ΔHbO. Directed PTE *t*-values for each ROI as a color-coded map for inactive subjects **(A–C)** and active subjects **(D–F)**. Hot (yellow-reds) and cold (light blue-dark blue) colors indicate information outflow and inflow, respectively. Arrows indicate statistically significant information flow between functional regions for inactive **(G–I)** and active subjects **(J–L)**. Black arrows (*p* < 0.05); Red arrows (*p* < 0.01). Eleven regions of interest (ROIs) were mapped: left and right frontopolar (lFP; rFP) (red), left and right pre-frontal cortex (lDLPFC; rDLPFC) (yellow), Broca’s area (green), left and right pre-motor cortex (lPMC; rPMC) (light blue), left and right primary motor and sensory cortical (lM1/S1; rM1/S1) areas (purple), and left and right sensory association cortex (lSAC; rSAC) (pink).

Information flow in the neurogenic frequency band is shown in Figures 4A–C (Supplementary Figures S2A–C for ΔHb) for inactive subjects and Figures 4D–F (Supplementary Figures S2D–F for ΔHb) for active subjects. At rest, inactive and active subject had dPTE sinks at the same ROIs, lFP and rFP (Figures 4A,D). These dPTE sinks persisted during the handgrip task while the number of dPTE sources increased between the 0–10 min and 11–20 min periods in both groups. In particular, inactive subjects had one dPTE source at rPMC in the first task period and none in the second period (Figures 4B,C). For active subjects, there were two sources at rPMC and lM1/S1 in the first period with two additional sources, at rDLPFC and lPMC, presenting in the second period (Figures 4E,F).

At the neurogenic frequency band, inactive subjects had more regions with significant net information flow than active subjects at resting state (inactive: 23, active: 10). However, active subjects had more significant regions with net information flow during the task at 0–10 min (inactive: 17, active: 25), and 11–20 min (inactive: 20, active: 26) (Figures 4G–L and Supplementary Figures S2G–L ΔHb). Additionally, both groups had several directed connections with higher statistical significance (*p* < 0.01) at the first half (inactive: 7, active: 3) and the second half (inactive: 7, active: 5) of the task. All but one of these connections were directed to the FP regions bilaterally (Figures 4G–L). At rest, inactive subjects had most ROIs connected to the FP regions bilateral (Figure 4G) whereas, active subjects’ statistically significant ROIs primarily connected to the lFP (Figure 4J). During the task, inactive subjects’ significant directional connections were more unilateral, favoring the contralateral hemisphere and primarily directed to the FP regions bilaterally (Figures 4H,I). In contrast, active subjects had more bilateral connections which were largely directed to the rFP, lFP, lDLPFC, and Broca’s area (Figures 4K,L).

#### Myogenic Frequency Band

**FIGURE 5:**
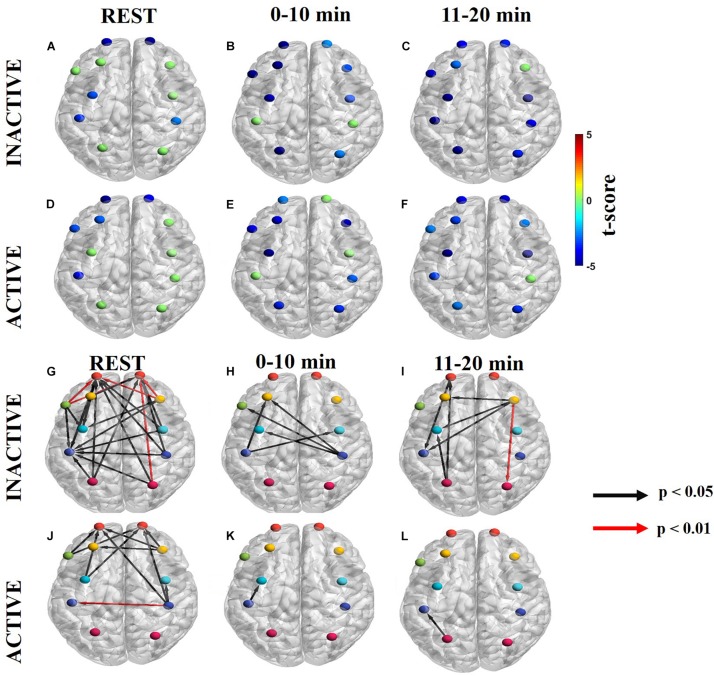
Significant dPTE and DC in the myogenic frequency band for inactive and active subjects during the handgrip task for ΔHbO. Directed PTE *t*-values for each ROI as a color-coded map for inactive subjects **(A–C)** and active subjects **(D–F)**. Hot (yellow-reds) and cold (light blue-dark blue) colors indicate information outflow and inflow, respectively. Arrows indicate statistically significant information flow between functional regions for inactive **(G–I)** and active subjects **(J–L)**. Black arrows (*p* < 0.05); Red arrows (*p* < 0.01). Eleven regions of interest (ROIs) were mapped: left and right frontopolar (lFP; rFP) (red), left and right pre-frontal cortex (lDLPFC; rDLPFC) (yellow), Broca’s area (green), left and right pre-motor cortex (lPMC; rPMC) (light blue), left and right primary motor and sensory cortical (lM1/S1; rM1/S1) areas (purple), and left and right sensory association cortex (lSAC; rSAC) (pink).

Information flow in the myogenic frequency band is shown in Figures 5A–C (Supplementary Figures S3A–C for ΔHb) for inactive subjects and Figures 5D–F (Supplementary Figures S3D–F for ΔHb) for active subjects for each task period. Notably, the myogenic frequency had no significant sources for both groups (Figures 5A–F). At rest, both groups had significant sinks at lFP, rFP, and lM1/S1 (Figures 5A,D). During the task, the number of ROIs with sinks increased from 9 to 10 in inactive subjects (Figures 5B,C) and from 8 to 10 in active subjects (Figures 5E,F).

At the myogenic frequency band, inactive subjects had more regions with significant net information flow than active subjects during the resting state (inactive: 21, active: 11), the 0–10 min (inactive: 5, active: 1), and the 11–20 min (inactive: 7, active: 1) task intervals (Figures 5G–L and Supplementary Figures S3G–L for ΔHb). During resting state, inactive subjects had five very significant (*p* < 0.01) directed connections originating from the rDLPFC, Broca’s area, and rSAC, all ending at the FP regions bilaterally (Figure 5G). In contrast, active subjects had only one very significant directed connection from rM1/S1 to lM1/S1 (Figure 5J). During the task, inactive subjects initially had net information outflow from M1/S1 bilaterally (Figure 5H), however, as the task progressed surrounding ROIs such as rDLPFC, and lSAC became information outflow sources directed at the lPMC and lM1/S1 (Figure 5I). In contrast, active subjects demonstrated lM1/S1 as a net information outflow to lPMC during the first half of the task (Figure 5K) but later transitioned as a net informational inflow from lSAC at the second half of the task (Figure 5L).

### Correlations Between SFC and FCV

**FIGURE 6:**
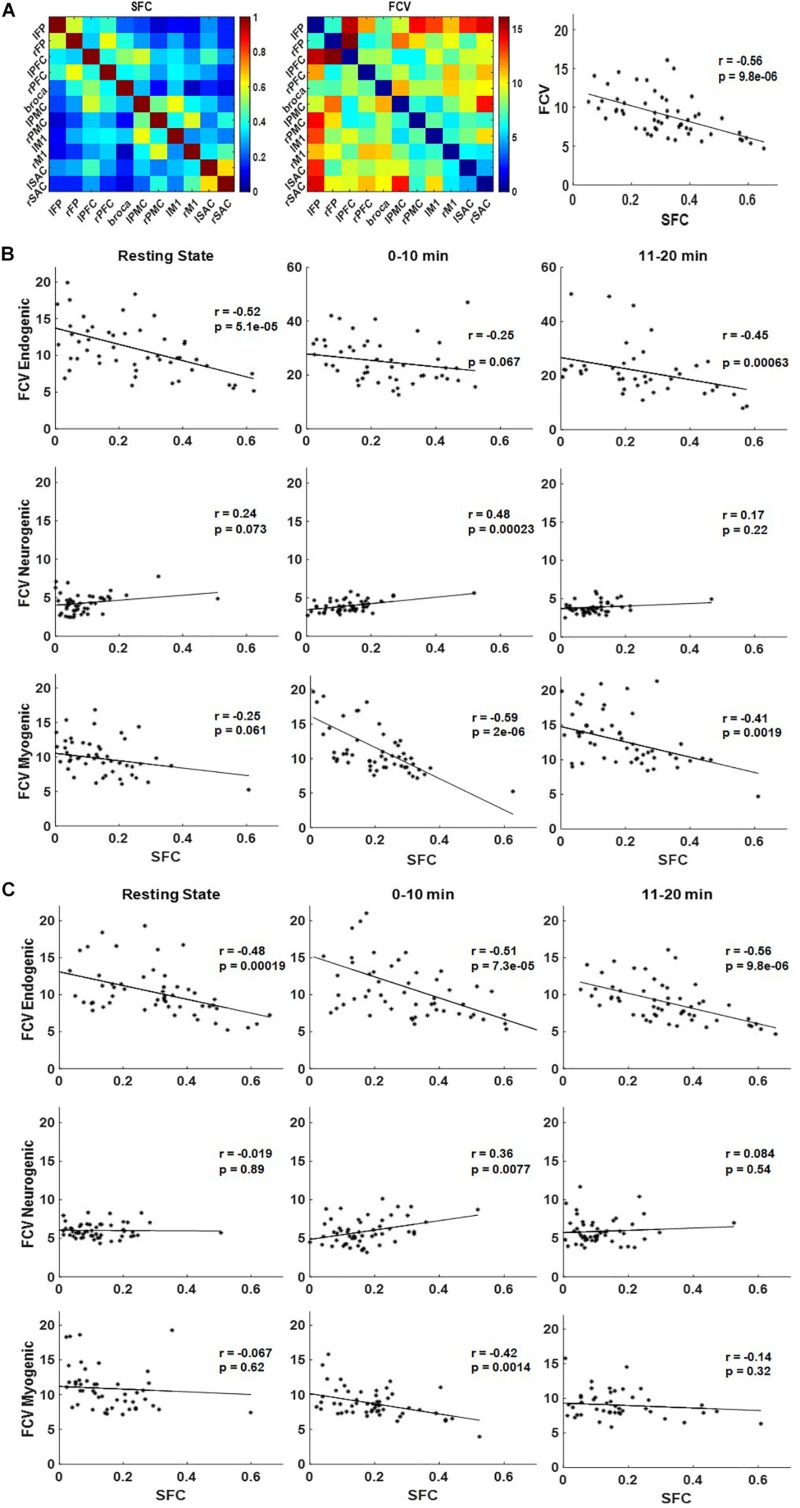
Pattern comparison between static functional connectivity (SFC) and functional connectivity variability (FCV) at endogenic, neurogenic, and myogenic frequencies for inactive and active subjects during the handgrip task. **(A)** A representative example of the group-averaged SFC matrix (left), the FCV matrix (middle), and the linear relationship between them (right). **(B)** Correlation plots between SFC and FCV for inactive subjects and **(C)** active subjects at endogenic (top), neurogenic (middle), and myogenic (bottom) frequencies during resting state (left), 0–10 min (middle), and 11–20 min (right) of the handgrip task.

Correlation patterns between SFC and FCV were also explored for each frequency band and subject group, before and during the handgrip task (Figure 6). A representative pattern comparison between group-level SFC and FCV matrices and the resulting linear correlation plot are shown in Figure 6A as an example of how the subsequent correlation plots were generated. Correlation plots for endogenic (top), neurogenic (middle), and myogenic (bottom) frequency bands are shown for inactive subjects in Figure 6B and active subjects in Figure 6C.

In the endogenic frequency band, quantitative correlation analysis revealed a strongly significant (*p* < 0.001) negative correlation between SFC and FCV at resting state and for the 11–20 min task interval for inactive subjects (Figure 6B, top row). Similarly, strong negative correlations were found for all task periods for active subjects (Figure 6C, top row). The correlation between SFC and FCV at 0–10 min for inactive subjects was not significant but was marginally close to the significance criterion of *p* = 0.05 (*p* = 0.07).

Correlation analysis was significant (*p* < 0.001) in the neurogenic frequency band both for inactive (Figure 6B, middle row) and active subjects at 0–10 min period only (Figure 6C, middle row). Pearson’s coefficient (*r*) values mostly indicated positive correlations, except for active subjects at resting state. Generally, the correlation trends were positive in contrast to the endogenic frequency band.

Quantitative correlation analysis at the myogenic frequency revealed significant (*p* < 0.01) negative correlation between SFC and FCV at 1–10 min and 11–20 min for inactive subjects (Figure 6B, bottom row) and at the 1–10 min period for active subjects (Figure 6C, bottom row). The correlation at the rest period for inactive subjects missed significance marginally (*p* = 0.06). The negative correlation trend in the myogenic frequency band was similar to that in the endogenic frequency band.

Overall, these plots illustrate a negative correlation trend in the endogenic and myogenic frequency bands and a positive correlation in the neurogenic frequency band, regardless of the physical activity levels of subjects. However, absolute *z*-values for inactive subjects were greater than active subjects for each frequency band, for both the positive and the negative trends, except for the 0–10 min and 11–20 min period in the endogenic frequency band.

## Discussion

Cerebral autoregulation is a multifactorial process of maintaining cerebral perfusion and brain tissue oxygenation against changes in arterial blood pressure that is challenged during exercise (Tzeng and Ainslie, 2014). The brain maintains relatively constant regional CBF (rCBF) through coordinated effort of endogenic, neurogenic and myogenic mechanisms that are known to be active in different hemodynamic frequency bands (Cipolla, 2009; Peterson et al., 2011; Tzeng and Ainslie, 2014; Peri-Okonny et al., 2015). The correspondence of each of these physiological mechanisms to distinct hemodynamic frequency bands enabled us to examine in this work how different fNIRS metrics could provide information about the interplay of these neurovascular coupling mechanisms during a fatiguing handgrip task.

Firstly, patterns of net information flow quantified by total dPTE-per-ROI (Hillebrand et al., 2016), calculated as the sum of all pair-wise contributions to each ROI, revealed which ROIs contributed to regulation (information sources) versus which were being regulated (information sinks) during the handgrip task (Huo et al., 2018). As the directionality of dominant contributions to net information flow is not evident in the total dPTE-per-ROI metric, the DC between ROIs was quantified also. The unidirectional connections indicate that one of the two ROIs serves as a functional source of coupling with a regulatory role over the connected sink region (Huo et al., 2018). While results for ΔHbO only were discussed above in detail, the corresponding ΔHb results were examined as well (Supplementary Material). In all, ΔHb signals yielded smaller dPTE values and fewer significant DC channel pairs due to smaller amplitudes and lower signal-to-noise ratio compared to ΔHbO signals, although the general trends were similar.

Furthermore, frequency band-based analyses were applied here to study the relationship between SFC, which measures coupling strength between ROIs and is known to increase with task performance (Jiang et al., 2012), and FCV, which reflects changes in spontaneous dynamic neural activity patterns between ROIs and relates to resource availability during demanding tasks (Li et al., 2015; Fong et al., 2019). The relationship between SFC and FCV was studied here for each hemodynamic frequency band to help understand their interdependence in the context of maintaining handgrip performance in the presence of fatigue. The latter was evident in the decline in %MVC (Figure 2) during a handgrip task. Our results showed differences between subject groups across all fNIRS metrics and hemodynamic frequency bands, suggesting that active subjects used different cortical activity strategies compared to inactive ones to maintain handgrip performance with increasing fatigue.

### Endogenic Frequency Band

Subjects with different physical activity levels displayed different dPTE and DC patterns in the endogenic (0.003–0.02 Hz) frequency band (Figure 3). Physical exercise increases CBF, as a result of elevated shear stress in the arterial walls which subsequently facilitates endogenic involvement in the regulation of rCBF (Nosarev et al., 2015). The cerebrovascular endothelium releases vasoactive mediators, including nitric oxide (NO) and endothelium-derived hyperpolarizing factor, diffusing into vascular smooth muscle, which contribute to CBF regulation through vasodilation (Cipolla, 2009; Peterson et al., 2011).

At rest, the dPTE and DC patterns together resemble the default mode network (DMN) for inactive subjects with either incoming (sinks) and outgoing (source) information flow at PMC, lM1/S1, Broca’s area, and rSAC. The DMN is highly engaged during rest and is involved in the emergence of spontaneous thought (van den Heuvel and Hulshoff Pol, 2010; Doucet et al., 2011, 2012; Raichlen et al., 2016). The DMN has previously been observed in the endogenic frequency for dPTE analysis (Cao et al., 2018b). and endogenic frequency specific FC maps (Bandettini and Bullmore, 2008). In this work active subjects did not exhibit any resting state dPTE network (indicating no net directional information flows) in the endogenic frequency band.

The handgrip task evoked significant dPTE and DC in the PFC and motor cortex in the endogenic frequency band, in particular for the inactive subjects. There was notable silencing of the ROIs associated with the DMN in dPTE and DC patterns once the task began, analogous to the DMN deactivation seen during tasks (Raichlen et al., 2016). For inactive subjects, the initial connection was from lPMC to Broca’s area. However, as fatigue increased the source became more unilateral in the left hemisphere at the motor cortex (lPMC and lM1/S1) and connected more to sinks in the PFC (FP and DLPFC). These observations suggest that motor regions (PMC and lM1/S1), which are involved in motor preparation and execution respectively (Frackowiak, 2004) regulated the DLPFC, which is associated with cognitive activity, planning, motivation during goal-driven tasks, and inhibiting/excitatory control during exercise (Semendeferi et al., 2001; Frackowiak, 2004; Marek and Dosenbach, 2018). Inactive subjects had notably more significant dPTE and DC than active subjects in the endogenic frequency band particularly during the first period. We speculate that active subjects had limited dPTE (lDLPFC and rSAC) and no significant DC until the second half of the task because regular exercise training enhances performance and the physically active subjects would presumably be more efficient earlier in the exercise task.

Strength of SFC is known to correlate with performance and increases with difficulty or effort in prior neuroimaging studies (Jiang et al., 2012; Rhee and Mehta, 2018). On the other hand, FCV signifies a cortical region that dynamically changes its connectivity strength with other regions so as to tap into more neuronal network resources while already established connections may fade in strength as fatigue sets in Fong et al. (2019). In the endogenic frequency, there is a negative correlation between SFC and FCV both for inactive (Figure 6B, top row) and active subjects (Figure 6C, top row). Several prior FCV studies have also found similarly negative correlations between these two metrics (Allen et al., 2014; Li et al., 2015; Fong et al., 2019). It has been suggested that task performance may depend on more stable (less variable) connectivity strength between regions involved in the regulation of the task (Fong et al., 2019). The more significant *r*-value (which indicate less variable connectivity) and lower %MCV reduction seen in active subjects (higher performance) compared to inactive ones is consistent with this interpretation.

Overall, the endogenic frequency band may be related to fatigue levels, meaning that higher fatigue would result in more endogenic regulation. We speculate that active subjects likely did not experience as much fatigue as inactive ones, as suggested by Figure 2, and supported by the difference in the amount of dPTE and DC between ROIs seen in Figures 3F,L.

### Neurogenic Frequency Band

Subjects with different physical activity levels displayed different dPTE and DC patterns in the neurogenic (0.02–0.04 Hz) frequency band. The neurogenic mechanism integrates the high metabolic demands of neuronal tissue during a task with the neurovascular unit (endothelial cells, perivascular nerves, and astrocytes abducted to cortical microvessels) to release vasoactive neurotransmitters as a means of regulating rCBF based on neuronal demands (Cipolla, 2009; Peterson et al., 2011).

At rest, the dPTE and DC patterns together resemble the DMN and fronto-parietal network (FPN) for inactive and active subjects with either incoming (sinks) and outgoing (source) information flow at PMC and SAC, which integrates sensory information and forms connections between sensory and motor areas, for the DMN and FP and SAC for the FPN. The FPN is highly integrated with other brain networks, like the DMN and motor network (MN), and is involved in coordinating behavior in a rapid, accurate, and flexible goal-driven manner (Doucet et al., 2011, 2012; Marek and Dosenbach, 2018), including planning of motor control (Raichlen et al., 2016). Our FPN similarity findings are supported by a neuroimaging study indicating that endurance athletes had more significant FC in the FPN than non-athletes, although this fMRI study focused on a frequency range that mostly included the neurogenic frequency band (Raichlen et al., 2016).

The handgrip task evoked significant dPTE and DC globally in the neurogenic frequency band for both active and inactive subjects. During the handgrip task, the FPN was still engaged as evident by dPTE and DC patterns. In FC studies, the FPN is known to activate during motor tasks, such as the fatiguing handgrip in this study (Urquhart et al., 2019), to provide a functional back-bone for rapid and flexible modulation of other brain networks, such as the MN (Marek and Dosenbach, 2018). Inactive and active subjects also had similar, persistent dPTE and DC patterns during the handgrip task although, active subjects had more significant dPTE ROIs and DC connections. The neurogenic frequency band reflects the cortical resources available during the task, thus suggesting that active subjects have more cortical resources and exercise-altered neuroplasticity than inactive subjects (Mueller et al., 2017). Lastly, in the neurogenic frequency band, there were greater numbers of significant DC connections during the task than at rest, for all subjects, similar to prior neuroimaging studies (Liu et al., 2003, 2007; Benwell et al., 2005; White et al., 2009; Mehta and Shortz, 2014; Zhiguo et al., 2016; Rhee and Mehta, 2018). During the handgrip task itself, the FP was a common functional sink in all subjects, but active subjects also involved Broca’s area. The FP region is associated with planning, cognitive branching, and monitoring importance of competing goal-driven tasks (Semendeferi et al., 2001; Frackowiak, 2004; Mansouri et al., 2017; Marek and Dosenbach, 2018). and Broca’s area is involved with producing language and inner speech (Perrone-Bertolotti et al., 2014). The latter is likely related to the silent expression of conscious thought to oneself during handgrip task performance in our study. In our prior work, we demonstrated that active subjects reported utilizing self-talk to motivate themselves during the task (Urquhart et al., 2019).

The neurogenic frequency band was the only frequency band to have a positive correlation between SFC and FCV for both inactive (Figure 6B, middle row) and active (Figure 6C, middle row) subjects. Prior neuroimaging studies also noted a positive correlation between exercise duration and increased hemodynamic signal variance (Benwell et al., 2005), and task performance and increasing neurogenic signal (Bosch et al., 2017). The non-significant *r*-value (which indicate more variable connectivity) and the %MCV reduction over time suggest that subjects were likely trying to tap into more cortical networks as they gradually fatigued. The significant *r*-values seen initially (0–10 min) in both groups support the notion that task performance may depend on more stable connectivity strength. As fatigue increased (11–20 min) both subject groups had more variable connectivity (i.e., non-significant *r*-value), possibly indicating an effort to recruit previously untapped cortical network resources.

Overall the neurogenic frequency band reflects the cortical resources available during the handgrip task (Boyle et al., 2008). Similar to FC, as physical effort for the task increased, the number of dPTE ROIs and DC connections increased as well before reducing again as fatigue increased. We hypothesize that active subjects had more dPTE ROIs and DC connections than inactive ones because they could recruit more cortical network resources, as enabled by exercise-related neuroplasticity.

### Myogenic Frequency Band

Subjects with different physical activity levels displayed different dPTE and DC patterns in the myogenic (0.04–0.15 Hz) frequency band. The myogenic regulatory mechanism plays an important role in stabilizing rCBF under differing physiological conditions, such as exercise (Cipolla, 2009). Exercise causes increased blood pressure resulting in depolarization of smooth muscle cell membrane and calcium influx and initiating the myogenic response, resulting in smooth muscles constricting during increased pressure (Cipolla, 2009; Peterson et al., 2011).

The resting state networks appeared to differ between inactive and active subjects in the myogenic frequency band based on dPTE and DC patterns. Inactive subjects’ functional sinks at FP and most significant DC pair-wise connections (red arrows) resemble the FPN. As previously mentioned, the FPN is often interconnected with other resting state networks, such as the MN to facilitate motor task control (Marek and Dosenbach, 2018). In contrast, the active subjects’ resting network was more reflective of the MN, with dPTE at M1/S1 and the most significant (red arrows) DC occurring between the M1/S1 areas as well as between the PMC areas bilaterally. The MN is a part of an extrinsic system of resting state networks that is typically driven by external sensory stimulation (Doucet et al., 2011). Active subjects appeared to have more active MN connectivity at rest than inactive subjects.

Inactive and active subjects also had different dPTE and DC patterns during the handgrip task in the myogenic frequency band. For inactive subjects, the lDLPFC was regulated by the M1/S1 during the first half (0–10 min). The M1/S1 sources are involved in motor execution, in particular during hand movement (Anwar et al., 2016; Jiang et al., 2012, Rhee and Mehta, 2018; Mehta and Shortz, 2014; Bajaj et al., 2014, Michely et al., 2018) which we hypothesize is why the DC connections were directed toward these motor planning sink regions. The DC to lDLPFC is interesting due to its association with an approach reaction toward a goal (Ernst et al., 2013), such as maintaining task performance. As fatigued worsened for inactive subjects, the DC to lDLPFC was regulated by the rDLPFC instead that, especially during prolonged exercise, in known to purposely inhibit bodily afferences that arise with physical fatigue to preserve mental effort during exercise maintenance (Rupp et al., 2013; Radel et al., 2017).

For active subjects, the lPMC was regulated by the lM1/S1 in the first half of the task. Like the inactive subjects, the lM1/S1 source is directed toward the motor planning associated PMC sink region. As fatigue worsened, the lM1/S1 became a net receiver of information by the lSAC source instead. Similarly, a prior EEG study demonstrated substantial location shifts of focal regions during a fatiguing handgrip task from anterior to posterior regions as a means to maintain task performance (Liu et al., 2007).

Lastly, inactive subjects had nearly significant negative correlations between FCV and SFC at rest and significant negative correlations during the task (Figure 6B, bottom row). Many FCV studies have found similarly negative correlations between these two metrics (Allen et al., 2014; Li et al., 2015; Fong et al., 2019). As previously mentioned, the significant *r*-values suggest that improved task performance requires less variable connectivity in the regulation of the task (Fong et al., 2019). While active subjects also exhibited negative correlations between FCV and SFC, these were not significant except during 0–10 min. Active subjects likely experienced lower fatigue levels at 11–20 min, which could contribute to the non-significant *r*-value (Figure 6C, bottom row). Mayer waves could also possibly confound myogenic frequency band results due to their overlapping frequency at about 0.1 Hz (Yücel et al., 2016).

Overall the myogenic frequency band reflects the blood supply to the ROIs that is regulated by the arterioles (Cipolla, 2009). We hypothesize that active subjects were able to dilate arterioles more efficiently (Green et al., 2012), and as a result they had improved blood supply to motor areas compared to inactive subjects. The increased availability of resources locally to the ROIs of active subjects controlling the motor task is a possible explanation for the lower number of DC pairs seen for this group relative to inactive subjects, whose ROIs had to recruit more resources from other locations while trying to maintain task performance.

## Limitations

There are several limitations to this study that should be addressed. This study did not incorporate multimodal monitoring of systemic hemodynamics, such as heart rate, blood pressure, and respiration, which could be used as regressors to improve fNIRS signals as well as provide information of the systemic physiology itself (Tachtsidis and Scholkmann, 2016). An alternative method would be to employ short-distance channels (<1 cm), which were not available in the commercial optode holder cap used, to remove by regression hemodynamic fluctuations that co-occur in the cortex as well as superficial scalp layers (Gagnon et al., 2012; Tachtsidis and Scholkmann, 2016). Although we used bandpass filters and PCA to remove physiological inferences and global hemodynamic fluctuations, there exists several other different computational methods including (i) independent component analysis, (ii) singular value decomposition (SVD) and Gaussian kernel smoothing, (iii) statistical correction methods, (iv) wavelet-based methods, or (v) a combination of these methods can be used for removal (Huppert et al., 2009; Jang et al., 2009; Tachtsidis and Scholkmann, 2016; Cao et al., 2018b; Duan et al., 2018; Klein and Kranczioch, 2019). Thus, a quantitative comparison using different global component removal methods is warranted in future studies.

## Conclusion

This study presents a direct comparison of differences in dynamic fNIRS metrics (dPTE, DC, SFC, FCV) between physically active and inactive subjects as they tried to maintain performance despite fatigue during a maximal effort task. The results of our study demonstrate how physical activity improves the neurovascular regulation mechanisms at the endogenic, neurogenic, and myogenic frequency bands. This modulation allowed for active subjects to maintain higher %MVC longer and delay fatigue onset. In future work we propose to expand these analyses to study subject populations of older age and impaired cardiovascular health.

## Data Availability Statement

The datasets generated for this study are available on request to the corresponding author.

## Ethics Statement

The studies involving human participants were reviewed and approved by the Institutional Review Board (IRB) at University of Texas at Arlington. The patients/participants provided their written informed consent to participate in this study.

## Author Contributions

EU, HL PF, and GA conceptualized and designed the study. EU acquired the data. EU and XW analyzed the data. EU and GA interpreted the results and wrote the manuscript. All authors read and approved the manuscript.

## Conflict of Interest

The authors declare that the research was conducted in the absence of any commercial or financial relationships that could be construed as a potential conflict of interest.

## Supplementary Material

FIGURE S1 Significant dPTE and DC in the endogenic frequency band for inactive and active subjects during the handgrip task for ΔHb. Directed PTE *t*-values for each ROI as a color-coded map for inactive subjects **(A–C)** and active subjects **(D–F)**. Hot (yellow-reds) and cold (light blue-dark blue) colors indicate information outflow and inflow, respectively. Arrows indicate statistically significant information flow between functional regions for inactive **(G–I)** and active subjects **(J–L)**. Black arrows (*p* < 0.05); Red arrows (*p* < 0.01). Eleven regions of interest (ROIs) were mapped: left and right frontopolar (lFP; rFP) (red), left and right pre-frontal cortex (lDLPFC; rDLPFC) (yellow), Broca’s area (green), left and right pre-motor cortex (lPMC; rPMC) (light blue), left and right primary motor and sensory cortical (lM1/S1; rM1/S1) areas (purple), and left and right sensory association cortex (lSAC; rSAC) (pink).

FIGURE S2 Significant dPTE and DC in the neurogenic frequency band for inactive and active subjects during the handgrip task for ΔHb. Directed PTE *t*-values for each ROI as a color-coded map for inactive subjects **(A–C)** and active subjects **(D–F)**. Hot (yellow-reds) and cold (light blue-dark blue) colors indicate information outflow and inflow, respectively. Arrows indicate statistically significant information flow between functional regions for inactive **(G–I)** and active subjects **(J–L)**. Black arrows (*p* < 0.05); Red arrows (*p* < 0.01). Eleven regions of interest (ROIs) were mapped: left and right frontopolar (lFP; rFP) (red), left and right pre-frontal cortex (lDLPFC; rDLPFC) (yellow), Broca’s area (green), left and right pre-motor cortex (lPMC; rPMC) (light blue), left and right primary motor and sensory cortical (lM1/S1; rM1/S1) areas (purple), and left and right sensory association cortex (lSAC; rSAC) (pink).

FIGURE S3 Significant dPTE and DC in the myogenic frequency band for inactive and active subjects during the handgrip task for ΔHb. Directed PTE *t*-values for each ROI as a color-coded map for inactive subjects **(A–C)** and active subjects **(D–F)**. Hot (yellow-reds) and cold (light blue-dark blue) colors indicate information outflow and inflow, respectively. Arrows indicate statistically significant information flow between functional regions for inactive **(G–I)** and active subjects **(J–L)**. Black arrows (*p* < 0.05); Red arrows (*p* < 0.01). Eleven regions of interest (ROIs) were mapped: left and right frontopolar (lFP; rFP) (red), left and right pre-frontal cortex (lDLPFC; rDLPFC) (yellow), Broca’s area (green), left and right pre-motor cortex (lPMC; rPMC) (light blue), left and right primary motor and sensory cortical (lM1/S1; rM1/S1) areas (purple), and left and right sensory association cortex (lSAC; rSAC) (pink).

TABLE S1 Spatial registration of fNIRS channel positions on a standard brain fMRI atlas. MNI coordinates displayed as mean (SD); r-right contralateral brain hemisphere; l- left ipsilateral brain hemispheres.

TABLE S2 Preprocessing input parameters used in Homer2.
