# Supplementary figures and images for: Differences in Net Information Flow and Dynamic Connectivity Metrics Between Physically Active and Inactive Subjects Measured by Functional Near-Infrared Spectroscopy (fNIRS) During a Fatiguing Handgrip Task
Source: Front Neurosci. 2020 Mar 10;14:167. doi: 10.3389/fnins.2020.00167 (PMC7076120; doi:10.3389/fnins.2020.00167)

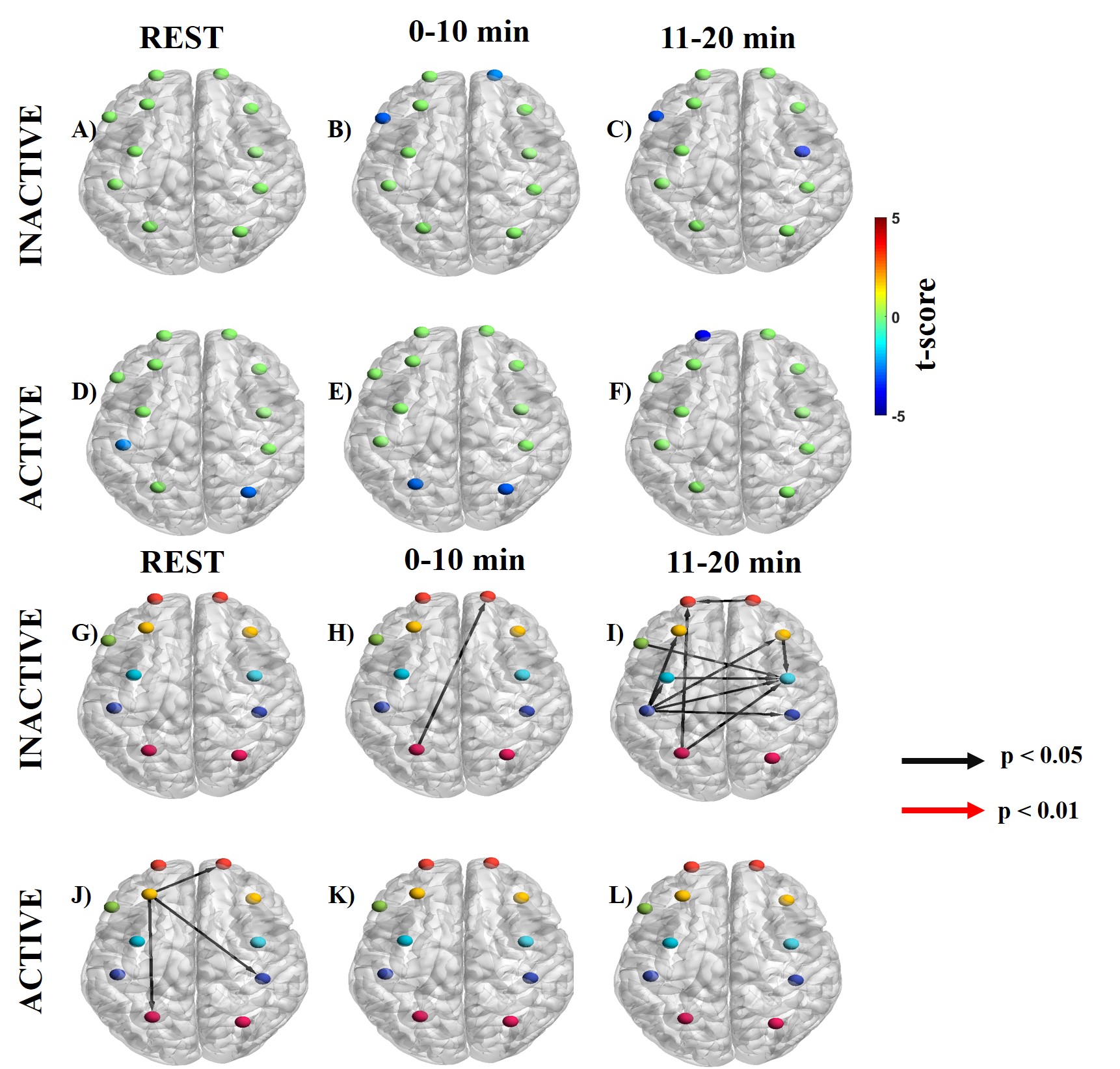

Supplement: FIGURE S1 — Significant dPTE and DC in the endogenic frequency band for inactive and active subjects during the handgrip task for ΔHb. Directed PTE t-values for each ROI as a color-coded map for inactive subjects (A–C) and active subjects (D–F). Hot (yellow-reds) and cold (light blue-dark blue) colors indicate information outflow and inflow, respectively. Arrows indicate statistically significant information flow between functional regions for inactive (G–I) and active subjects (J–L). Black arrows (p < 0.05); Red arrows (p < 0.01). Eleven regions of interest (ROIs) were mapped: left and right frontopolar (lFP; rFP) (red), left and right pre-frontal cortex (lDLPFC; rDLPFC) (yellow), Broca’s area (green), left and right pre-motor cortex (lPMC; rPMC) (light blue), left and right primary motor and sensory cortical (lM1/S1; rM1/S1) areas (purple), and left and right sensory association cortex (lSAC; rSAC) (pink). [file Image_1.JPEG]

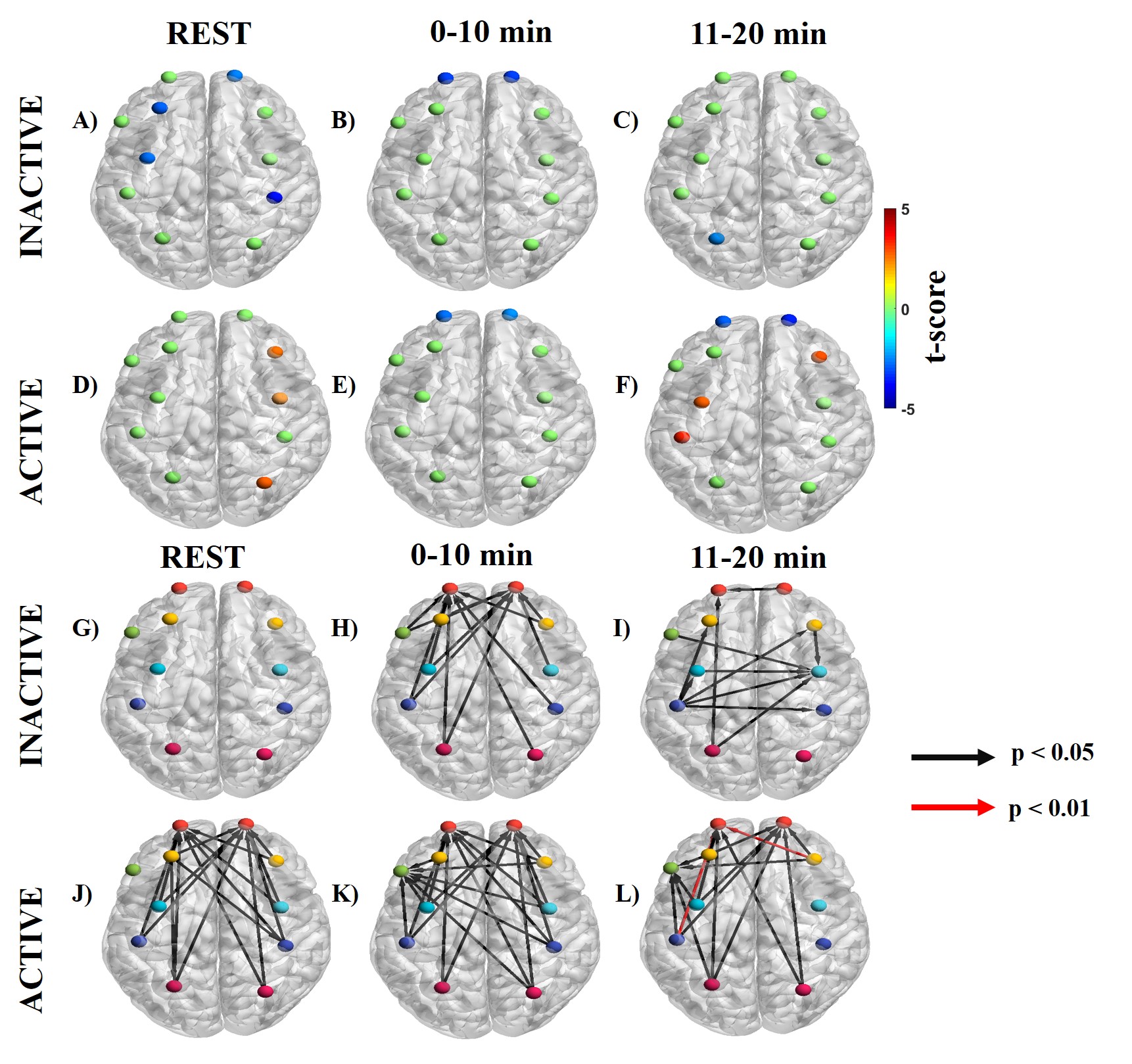

Supplement: FIGURE S2 — Significant dPTE and DC in the neurogenic frequency band for inactive and active subjects during the handgrip task for ΔHb. Directed PTE t-values for each ROI as a color-coded map for inactive subjects (A–C) and active subjects (D–F). Hot (yellow-reds) and cold (light blue-dark blue) colors indicate information outflow and inflow, respectively. Arrows indicate statistically significant information flow between functional regions for inactive (G–I) and active subjects (J–L). Black arrows (p < 0.05); Red arrows (p < 0.01). Eleven regions of interest (ROIs) were mapped: left and right frontopolar (lFP; rFP) (red), left and right pre-frontal cortex (lDLPFC; rDLPFC) (yellow), Broca’s area (green), left and right pre-motor cortex (lPMC; rPMC) (light blue), left and right primary motor and sensory cortical (lM1/S1; rM1/S1) areas (purple), and left and right sensory association cortex (lSAC; rSAC) (pink). [file Image_2.JPEG]

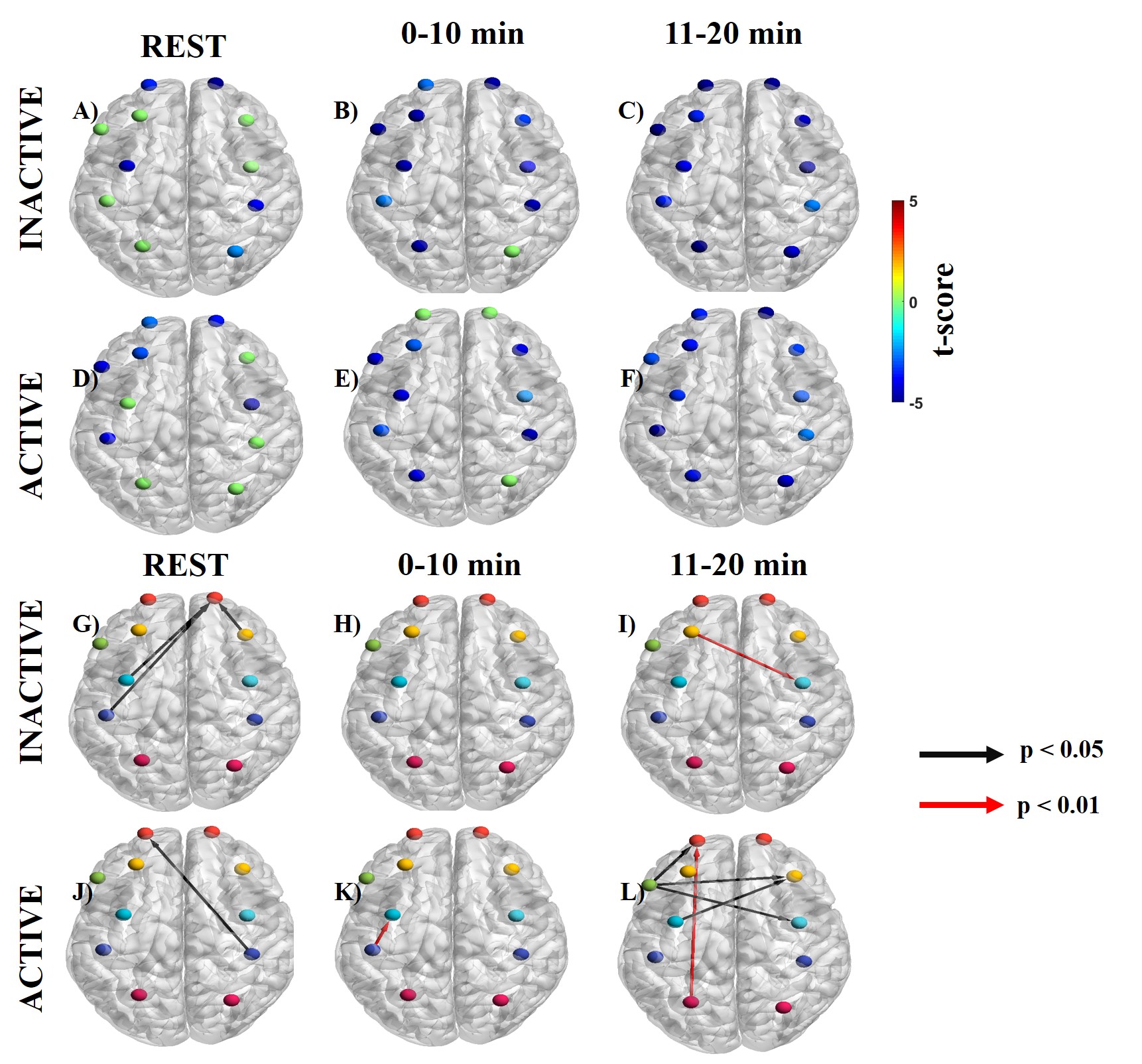

Supplement: FIGURE S3 — Significant dPTE and DC in the myogenic frequency band for inactive and active subjects during the handgrip task for ΔHb. Directed PTE t-values for each ROI as a color-coded map for inactive subjects (A–C) and active subjects (D–F). Hot (yellow-reds) and cold (light blue-dark blue) colors indicate information outflow and inflow, respectively. Arrows indicate statistically significant information flow between functional regions for inactive (G–I) and active subjects (J–L). Black arrows (p < 0.05); Red arrows (p < 0.01). Eleven regions of interest (ROIs) were mapped: left and right frontopolar (lFP; rFP) (red), left and right pre-frontal cortex (lDLPFC; rDLPFC) (yellow), Broca’s area (green), left and right pre-motor cortex (lPMC; rPMC) (light blue), left and right primary motor and sensory cortical (lM1/S1; rM1/S1) areas (purple), and left and right sensory association cortex (lSAC; rSAC) (pink). [file Image_3.JPEG]
