# Supplementary material for: Differences in Net Information Flow and Dynamic Connectivity Metrics Between Physically Active and Inactive Subjects Measured by Functional Near-Infrared Spectroscopy (fNIRS) During a Fatiguing Handgrip Task
Source: Front Neurosci. 2020 Mar 10;14:167. doi: 10.3389/fnins.2020.00167 (PMC7076120; doi:10.3389/fnins.2020.00167)
Supplement: TABLE S1 — Spatial registration of fNIRS channel positions on a standard brain fMRI atlas. MNI coordinates displayed as mean (SD); r-right contralateral brain hemisphere; l- left ipsilateral brain hemispheres. [file Table_1.pdf]

Table S1 Spatial registration of fNIRS channel positions on a standard brain fMRI atlas. MNI coordinates displayed as mean (SD); r-right contralateral brain hemisphere; l- left ipsilateral brain hemispheres.

CHANNEL		*MNI*		*REGION OF*	**BRODMANN**
	X	*Y*	*Z*	*INTEREST (ROI)*	**AREA (BA)**
1	-28.92(5.02)	66.44(1.93)	5.92(10.45)	lFP	BA10
2	-10.25(4.51)	71.27(2.29)	9.36(10.64)	lFP	BA10
3	15.74(2.76)	71.79(1.87)	9.95(11.07)	rFP	BA10
4	35.32(4.11)	64.89(2.91)	5.12(10.03)	rFP	BA10
5	-18.92(3.72)	68.30(2.73)	16.80(10.55)	lFP	BA10
7	24.85(3.94)	67.33(3.88)	17.24(9.80)	rFP	BA10
8	-10.14(3.90)	65.39(5.04)	28.02(10.45)	lFP	BA10
9	15.14(2.34)	65.50(5.42)	28.32(9.24)	rFP	BA10
10	-18.70(4.14)	58.42(6.75)	34.80(10.06)	lDLPFC	BA9
12	23.84(4.12)	57.20(7.52)	35.33(8.66)	rDLPFC	BA9
13	-44.86(4.27)	45.98(6.40)	22.55(9.36)	Broca's	BA44/45
14	-29.03(5.33)	49.56(8.06)	37.27(9.58)	lDLPFC	BA9
15	-9.38(2.74)	52.12(9.01)	46.03(8.77)	lDLPFC	BA9
16	14.47(2.39)	51.38(9.31)	46.27(8.47)	rDLPFC	BA9
17	33.59(4.55)	48.26(8.68)	37.98(7.90)	rDLPFC	BA9
18	48.38(3.69)	45.52(7.43)	22.92(8.17)	rDLPFC	BA9
19	-52.56(4.03)	33.44(7.80)	22.02(5.60)	Broca's	BA44/45
20	-39.89(4.55)	38.27(9.44)	39.21(9.17)	lDLPFC	BA9
21	-19.52(3.54)	40.94(10.90)	51.24(9.18)	lDLPFC	BA9
23	23.44(3.81)	40.15(10.54)	52.16(7.06)	rDLPFC	BA9
24	41.97(7.65)	36.82(9.38)	40.21(7.67)	rDLPFC	BA9
25	54.21(7.13)	32.68(8.64)	23.36(7.57)	Broca's	BA44/45
27	-58.54(3.43)	19.56(9.53)	21.71(7.33)	Broca's	BA44/45
28	-48.61(4.40)	25.98(9.87)	39.86(7.89)	Broca's	BA44/45
29	-31.88(5.64)	28.91(11.57)	53.58(8.18)	lDLPFC	BA9
32	33.44(6.50)	28.23(11.01)	54.55(5.99)	rDLPFC	BA9
33	50.08(5.45)	24.61(10.67)	41.23(6.81)	rDLPFC	BA9
38	-56.91(4.65)	10.29(11.67)	37.30(6.35)	lPMC	BA6
39	-42.89(4.48)	16.08(12.82)	5427(5.84)	lDLPFC	BA9
43	44.30(5.84)	14.72(13.02)	54.74(5.04)	rDLPFC	BA9
45	66.92(2.55)	1.39(9.31)	18.61(7.65)	rPMC	BA6
49	-51.35(4.99)	1.94(14.33)	52.29(4.78)	lPMC	BA6
50	-33.21(8.22)	5.89(15.35)	65.29(4.91)	lPMC	BA6
51	-13.64(4.83)	7.74(15.97)	71.09(6.52)	lPMC	BA6
52	13.56(6.13)	6.86(16.50)	72.09(4.26)	rPMC	BA6
54	53.55(5.44)	-1.08(13.36)	52.15(4.31)	rDLPFC	BA9
55	66.23(3.48)	-7.32(10.61)	34.15(5.19)	rPMC	BA6
60	-44.47(3.17)	-8.64(15.74)	61.53(3.68)	lPMC	BA6
61	-22.38(3.13)	-8.42(17.48)	73.18(2.64)	lPMC	BA6
63	23.73(2.47)	-9.14(16.25)	73.74(8.32)	rPMC	BA6
64	47.03(3.30)	-12.36(14.16)	62.18(2.84)	rPMC	BA6
65	63.67(2.77)	-19.62(11.49)	46(4.08)	rPMC	BA6

70	-54.95(2.56)	-26.30(14.18)	55.56(3.49)	lS1	BA1/2/3
71	-35.54(2.76)	-23.18(16.20)	70.86(2.40)	lM1	BA4
72	-13.97(1.75)	-20.91(16.47)	77.76(1.37)	lM1	BA4
73	14.26(1.47)	-21.55(15.61)	77.77(0.88)	rPMC	BA6
74	36.65(3.68)	-23.80(17.56)	71.47(1.44)	rM1	BA4
75	56.12(457)	-29.95(12.78)	56.02(2.95)	rS1	BA1/2/3
76	67.41(3.16)	-49.94(63.79)	38.03(5.91)	lS1	BA1/2/3
81	-46.74(3.49)	-37.18(14.07)	63.06(4.34)	lS1	BA1/2/3
82	-23.38(5.10)	-36.33(15.76)	74.06(3.00)	lM1	BA4
84	24.74(3.07)	-37.65(14.31)	73.61(6.53)	rM1	BA4
85	47.24(5.01)	-39.26(13.27)	62.92(3.91)	rS1	BA1/2/3
95	34.62(4.74)	-53.02(11.79)	67.73(5.53)	rS1	BA1/2/3
101	-24.77(3.71)	-63.60(10.99)	66.97(8.40)	lSAC	BA7
103	25.09(3.98)	-63.61(10.25)	65.98(11.02)	rSAC	BA7
107	-36.12(5.15)	-74.42(9.73)	51.08(10.61)	lSAC	BA7
108	-16.06(2.76)	-72.80(18.20)	60 (11.26)	lSAC	BA7
109	14.91(4.26)	-75.30(8.57)	60.33(11.14)	rSAC	BA7
110	33.50(5.72)	-72.80(17.21)	51.23(9.97)	rSAC	BA7
