# Supplementary material for: Differences in Net Information Flow and Dynamic Connectivity Metrics Between Physically Active and Inactive Subjects Measured by Functional Near-Infrared Spectroscopy (fNIRS) During a Fatiguing Handgrip Task
Source: Front Neurosci. 2020 Mar 10;14:167. doi: 10.3389/fnins.2020.00167 (PMC7076120; doi:10.3389/fnins.2020.00167)
Supplement: TABLE S2 — Preprocessing input parameters used in Homer2. [file Table_2.pdf]

Table S2. Preprocessing input parameters used in Homer2

Function	Input parameters	
hmrIntensity2OD		
enPCAFilter	nSV = 2	
hmrBandPassFilt	Endogenic	hpf = 0.003<br>lpf = 0.02
	Neurogenic	hpf = 0.02<br>lpf = 0.04
	Myogenic	hpf = 0.04<br>lpf = 0.15
hmrOD2Conc	6 6 6	
